# Machine Learning Identifies Chronic Low Back Pain Patients from an Instrumented Trunk Bending and Return Test

**DOI:** 10.3390/s22135027

**Published:** 2022-07-03

**Authors:** Paul Thiry, Martin Houry, Laurent Philippe, Olivier Nocent, Fabien Buisseret, Frédéric Dierick, Rim Slama, William Bertucci, André Thévenon, Emilie Simoneau-Buessinger

**Affiliations:** 1 LAMIH, CNRS, UMR 8201, Université Polytechnique Hauts-de-France, 59313 Valenciennes, France; emilie.simoneau@uphf.fr; 2 CHU Lille, Université de Lille, 59000 Lille, France; andre.thevenon@univ-lille.fr; 3 CeREF Technique, Chaussée de Binche 159, 7000 Mons, Belgium; buisseretf@helha.be (F.B.); frederic.dierick@rehazenter.lu (F.D.); 4 Centre de Recherche FoRS, Haute-Ecole de Namur-Liège-Luxembourg (Henallux), Rue Victor Libert 36H, 6900 Marche-en-Famenne, Belgium; martin.houry@henallux.be (M.H.); laurent.philippe@henallux.be (L.P.); 5 PSMS, Université de Reims Champagne Ardenne, 51867 Reims, France; olivier.nocent@univ-reims.fr (O.N.); william.bertucci@univ-reims.fr (W.B.); 6 Service de Physique Nucléaire et Subnucléaire, UMONS Research Institute for Complex Systems, Université de Mons, Place du Parc 20, 7000 Mons, Belgium; 7 Centre National de Rééducation Fonctionnelle et de Réadaptation–Rehazenter, Laboratoire d’Analyse du Mouvement et de la Posture (LAMP), Rue André Vésale 1, 2674 Luxembourg, Luxembourg; 8 Faculté des Sciences de la Motricité, UCLouvain, Place Pierre de Coubertin 1, 1348 Ottignies-Louvain-la-Neuve, Belgium; 9 LINEACT Laboratory, CESI Lyon, 69100 Villeurbanne, France; rsalmi@cesi.fr

**Keywords:** artificial intelligence, machine learning, inertial measurement unit—IMU, movement complexity, sample entropy, trunk flexion

## Abstract

Nowadays, the better assessment of low back pain (LBP) is an important challenge, as it is the leading musculoskeletal condition worldwide in terms of years of disability. The objective of this study was to evaluate the relevance of various machine learning (ML) algorithms and Sample Entropy (SampEn), which assesses the complexity of motion variability in identifying the condition of low back pain. Twenty chronic low-back pain (CLBP) patients and 20 healthy non-LBP participants performed 1-min repetitive bending (flexion) and return (extension) trunk movements. Analysis was performed using the time series recorded by three inertial sensors attached to the participants. It was found that SampEn was significantly lower in CLBP patients, indicating a loss of movement complexity due to LBP. Gaussian Naive Bayes ML proved to be the best of the various tested algorithms, achieving 79% accuracy in identifying CLBP patients. Angular velocity of flexion movement was the most discriminative feature in the ML analysis. This study demonstrated that: supervised ML and a complexity assessment of trunk movement variability are useful in the identification of CLBP condition, and that simple kinematic indicators are sensitive to this condition. Therefore, ML could be progressively adopted by clinicians in the assessment of CLBP patients.

## 1. Introduction

Low back pain (LBP) is the leading cause of a high number of years lived with disability worldwide. In both 10–24 and 50–74-year-old age groups, LBP is typically responsible for the loss of an entire year of full health [1]. A better understanding of this musculoskeletal condition and its complexity is therefore essential for clinicians involved in the care of patients with LBP.

LBP, especially chronic LBP (CLBP), leads to a fear of movement and causes patients to limit their activities of daily living and social participation to avoid pain [2,3]. The sedentary lifestyle of LBP patients is an exacerbating factor and leads to chronicity [4,5]. A better understanding of the relationship between the kinematics of the lumbo–pelvic–hip complex and LBP is currently of high importance. The evaluation of these movements can potentially be performed easily and inexpensively with one or more low-complexity devices [6], such as inertial sensors, also called inertial measurement units (IMUs) [7,8,9,10,11]. Low complexity can be understood as ready-to-use, which is a necessary feature in clinical practice. Information from the IMU time series can be obtained using various methods, including machine learning (ML) algorithms [12,13,14,15,16,17,18]. Our study focused on IMU-based testing of lumbo–pelvic–hip complex movements and ML-based algorithm analysis of CLBP patients and non-LBP (NLBP) subjects. The power of current computers and commercially available equipment allows ML to be increasingly affordable; hence it seemed logical to include it in the development of a clinical test to assess LBP.

An example of interest for the present study is the ability of a single IMU to measure the variability of repetitive trunk bending and return (b&r) movements by computing the sample entropy (SampEn) [19] from the different time series recorded in healthy subjects [20]. In the latter study [20], it was shown that 50 repetitions of trunk b&r movements could provide kinematic data that allow for the accurate computation of SampEn from the six time series recorded by a single IMU and, therefore, may be used to assess movement complexity [21]. We hypothesize that such a b&r test can be used to investigate LBP and, if possible, discriminate between the presence and absence of LBP in individuals. In the present study, we extended the results of [20] in several directions: (1) increasing the number of IMU from one to three; (2) using SampEn measurements in combination with ML methods to analyze the recorded time series; (3) including patients with CLBP in the population of NLBP subjects previously included in [20]. We will now address these three points.

1. The use of a set of three IMUs should provide more information about the b&r test and allows the whole lumbopelvic–hip complex to be examined. Some authors placed the IMUs at L2 and S2 vertebrae to measure low lumbar flexion–extension movements [22], while others chose T10-12 and S2 to be able to measure the local dynamic stability, coordination, and variability of the lumbar spine in repeated flexion–extension movements [23]. We chose T12 and S2 so to consider the entire lumbar spine for a given movement. Differences between the time series of different sensors can provide information about the relative angular velocity and acceleration between two anatomical landmarks. Typically, for sagittal plane angular velocities, the difference between the data from a sensor placed at the twelfth thoracic vertebra and a sensor placed at the second sacral vertebra should provide an estimate of the lumbar angular velocity. From a clinical perspective, the use of three IMUs placed at different points may help to identify the most relevant location for a single IMU, which may be useful when time constraints apply.
2. Two main techniques were used in this study. The first is a standard statistical analysis, which consists of computing the SampEn from the IMUs time series in two groups—NLBP subjects and CLBP patients—and comparing them. The second technique is ML, which has been used for about 40 years in the study of LBP [13], especially in the field of medical imaging and clinical data analysis for diagnostic and decision-making purposes [14,15]. Note that SampEn will be part of the data used by ML to identify CLBP patients.
3. We believe that a first step toward integrating the clinical interpretation of a test, such as the b&r test, into a biopsychosocial model must be to examine its ability to discriminate between CLBP patients and NLBP subjects. A recent study using a particular supervised ML algorithm (Support Vector Machine, SVM) to analyze IMU data has already shown that a kinematic test of the lumbar spine is able to discriminate NLBP subjects from LBP patients and classify them according to their risk of chronicity, i.e., between high risk and medium to low risk, with an accuracy of >75% [12]. Moreover, it has been shown in [24] that SVM can detect neck pain from rotational head movements with an accuracy of 82%. These last two studies show that a diagnostic analysis using ML algorithms supplied with kinematic parameters is a promising way to investigate these spinal conditions further. Our work focuses on the kinematic signature of patients suffering from CLBP and, more specifically, on the complexity of their variability during repetitive movements of the trunk along the lines of [20].

The main question addressed by this study is: “Can an instrumented b&r test identify CLBP patients by resorting to ML algorithms?” Our hypothesis is that algorithms such as SVM can accurately discriminate CLBP patients from NLBP subjects using raw data from three IMUs and SampEn values.

## 2. Materials and Methods

### 2.1. Population

Data were collected from a group of CLBP patients and from a matched group of healthy NLBP subjects. The study protocol was approved by the Intercommunale de Santé Publique du Pays de Charleroi Ethics Committee (ISPPC/OM008) under the number B325-2020-43666 and complied with the Helsinki Declaration on the Ethical Principles for Medical Research Involving Human Beings. All of the patients and subjects received an information sheet explaining the purpose of the study and gave informed consent before participation.

Twenty CLBP patients were recruited on a voluntary basis between the 3 November and the 1 December 2020 from the pool of patients treated for CLBP at the University Hospital of Charleroi (CHUC) in the “Sport Santé” department in Monceau-sur-Sambre, Belgium. The inclusion criteria were: the presence of LBP diagnosed by a physician and lasting longer than three months, ability to perform three trunk flexions with the lower limbs extended, aged between 18 and 65 years, body mass index (BMI) between 18 and 35 kg·m^−2^, and a pain score of less than 8/10 on a verbal Numerical Rating Scale (NRS) the day of the test. The exclusion criteria were: the presence of tumor, fracture, neurological signs (loss of strength and/or sensitivity), decrease or abolition of reflexes, and presence of pain of neuropathic origin evaluated by a positive DN4 (“diagnostic de Douleur Neuropathique”, i.e., neuropathic pain diagnostic) questionnaire [25], recent trauma, surgery at the spinal level, vertigo and balance disorders due to positional changes, musculoskeletal disorders in another region that could interfere with the b&r test, or systemic and metabolic disorders.

Twenty healthy NLBP subjects were recruited on a voluntary basis between 1 December and 15 December 2020 from CHUC staff or their personal acquaintances. They were matched with the CLBP patients using the following criteria: age, BMI, and physical activity level. For these subjects, the exclusion criteria were: the presence of LBP in the past year, history of spinal surgery, musculoskeletal disorders in another region that could interfere with the b&r test, systemic and metabolic disorders, and the use of analgesics.

### 2.2. Protocol, Data Collection and Preprocessing

**Figure 1:**
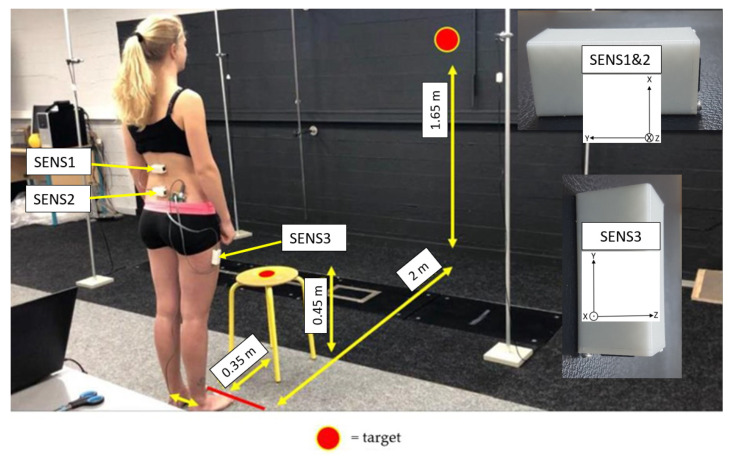
NLBP subject in starting position for the b&r test. The targets on the stool and on the wall in front of the subject are shown (red points). A zoom on the sensors is shown in the inset of the figure (on the right). The respective coordinate system (X, Y, Z) used is shown for SENS1&2 and for SENS3.

The general characteristics were collected from all participants: gender, age, BMI, and physical activity level were collected through the Global Physical Activity Questionnaire (GPAQ) [26]. Two experienced examiners (physiotherapists) were involved in the protocol. A first examiner (examiner #1) gave standardized instructions to each participant and showed a video demonstrating how to perform the b&r test. The same examiner (#1) cleaned the skin with cotton wool and ether at the sites where the three IMUs were taped. These were attached with hypoallergenic double-sided adhesive tape (Figure 1): Sensor #1 (SENS1) on the opposite to the spinous process of the twelfth thoracic vertebra (T12); Sensor #2 (SENS2) opposite to the second sacral vertebra (S2) on a horizontal line connecting the posterior-superior iliac spines; and Sensor #3 (SENS3) on the lateral side of the thigh, 10 cm below the greater trochanter.

Each participant was then placed in a standing position with their arms by their sides, as shown in Figure 1. To perform the b&r test, the participants were instructed to bend the trunk forward (flexion) without flexing their lower limbs and to touch a target in the center of a stool with the fingertips of both hands, followed by an immediate trunk extension to return to the starting position while focusing on a target in front of them (Figure 1). This movement was repeated continuously, as quickly and comfortably as possible. The test was performed once and lasted 70 s. The participants were allowed to stop the test before the end of the 70 s if they felt unable to continue; however, in this case, they were then excluded from the study. The examiner was present near the participant during the test but gave no verbal instructions.

A second examiner (examiner #2) recorded the data from the IMUs on a computer located less than 3 m from the patient or subject, with the IMUs connected to the computer via a cable [27]. Examiner #2 informed the participant of the time remaining every 10 s. The computer screen was visible to neither examiner #1 nor the participant. The data were stored on the computer’s hard disk drive for later analysis.

**Figure 2:**
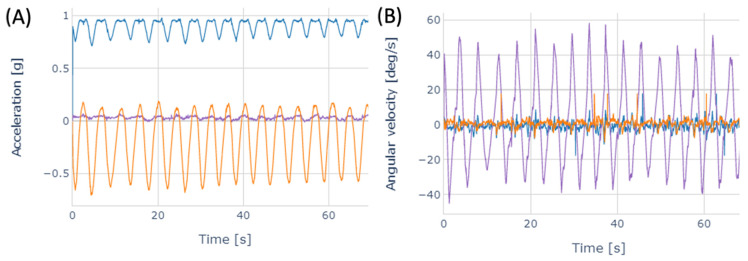
Typical traces of (**A**) Acc X (blue), Acc Y (purple), and Acc Z (orange) and (**B**) Gyr X (blue), Gyr Y (purple), and Gyr Z (orange) time series recorded with SENS1 during a b&r test in a healthy NLBP subject.

The IMUs used are part of a homemade system called DYSKIMOT (for Motor Dyskinesia), which has been presented in detail previously in [27]. The DYSKIMOT system software (v. 3.1) recorded the data from the three IMUs with a sampling frequency of 100 Hz. The system is based on commercial IMUs (LSM9DS1, SparkFun Electronics, Niwot, CO, USA) equipped with a triaxial accelerometer, gyroscope, magnetometer, and thermometer. Time series of accelerations (Acc X, Y, Z) along each of the 3 axes of each sensor and angular velocities (Gyr X, Y, Z) were recorded. The DYSKIMOT software also computed the angular accelerations (AccA X, Y, Z) in real-time by numerical differentiation (method) and the corrected angles (AngC X, Y, Z) by numerical integration and linear drift subtraction of the angular velocities. Typical traces are shown in Figure 2. The DYSKIMOT IMU casing was 3D printed using PLA (Polylactic Acid), the most widely used polymer in 3D printing. Other polymers may have been used, such as ABS (Acrylonitrile butadiene styrene) or TPU (Thermoplastic polyurethane). The latter choice made it possible to produce flexible parts that would presumably be more comfortable for the user.

As in [2], we considered the first 10 s of the time series as the warm-up period, and any data collected after 70 s were omitted from analyses. Therefore, only data between 10 and 70 s were used for the analysis; all of the time series lasted 1 min.

### 2.3. SampEn and Complexity Factors

An R (v 4.1.0, R Core Team, Auckland, New Zealand) routine was developed to calculate SampEn for all recorded time series. In accordance with Yentes [19], we used the following parameters in the algorithm (R-Studio) for calculating the SampEn: vector length m = 2, tolerance r = 0.2 SD and the number of points in the data series N = 6000. The details can be found in [20]. The values obtained from Gyr and Acc, respectively, were referred to as SampEn Gyr X, Y, Z and SampEn Acc X, Y, Z.

In the case of the b&r test, the Gyr Y time series should have considerable practical implications since it follows the direction of the performed movement. Therefore, specific computations were performed for this time series. The Gyr Y of SENS2 was subtracted from the same time series of SENS1 (Gyr Y) and SENS3 (−Gyr Z; SENS3 has a different orientation, see Figure 1). SampEn was computed for the resulting time series. The calculation of SampEn using these data should provide a relevant estimation of the complexity of angular velocity variations in lumbar and hip -flexion during the b&r test. The SampEn values obtained in this way were termed the lumbar flexion complexity factor (LCF) and the hip flexion complexity factor (HCF), and together formed what we called complexity factors (CF).

Statistical comparisons between all SampEn and CF in CLBP patients and NLBP subjects were performed with a t-test or with a Mann–Whitney rank sum when the Shapiro–Wilk normality test failed. The mean, SD, standard error of measurement ($SEM=\frac{SD}{\sqrt{N}}$, where N is the sample size), 95% confidence interval (CI denotes half-width), and minimum detectable change ($MDC=1.96\sqrt{2}\;SEM$) were calculated for the differences in parameter values between the two groups. Statistical tests were performed with SigmaPlot software (v. 14.0, Systat Software, San Jose, CA, United States of America) with a significance level of 5%.

### 2.4. Machine Learning Analysis

#### 2.4.1. Data Segmentation

The first way to apply ML is to use and analyze a given time series as a whole. A second way is to divide the time series into cycles and use them as independent data related to a particular participant. Since we have explored both options, further details on segmentation into cycles are provided below.

The different b&r cycles can be clearly seen in Figure 2. Each cycle corresponds to the repetition of b&r trunk movements carried out by a participant. The start and end of each cycle were determined by identifying the successive minima of the Acc Z time series for SENS1, i.e., the time series with the highest signal-to-noise ratio.

The segmentation algorithm, which divides the time series into distinct cycles, consists of five steps: (1) averaging the original Acc Z time series using a rolling centered window (width: 25 points or 0.25 s); (2) calculating the global amplitude and the exact value of the threshold (40% of the amplitude above the minimum); (3) extracting data below this threshold to avoid the processing of local minimum above this threshold; (4) detecting the minima and computing the cycle limits as the average position of two consecutive minima; (5) finally, all cycles were normalized by linear interpolation to 450 points, which is the maximum number of points for a cycle in our data set. The last 450 points were referred to as “itime”.

In the Results section, ML analysis based on 1-min time series is referred to as “whole sequences”, and ML analysis based on individual cycles is referred to as “cycle segmentation”.

#### 2.4.2. Discrimination of NLBP and CLBP Participants

The discriminating power of classification by the ML algorithm was assessed by computing accuracy and Area Under Curve (AUC) values for binary classification. Several classification algorithms were used, all belonging to the supervised ML, as each participant was labeled with either NLBP or CLBP condition. The ML algorithms studied were Linear Support Vector Machines (Linear SVM), Non-linear Support Vector Machine Radial Basis Function (SVM RBF), Random Forest (RF), Gaussian naive Bayes (GaussianNB), K-neighbors (KNN), Adaptive Boosting (AdaBoost), and Decision Tree (DT) [28]. The grid-search method [29] was used to optimize the hyperparameters for each ML algorithm. The hyperparameters are listed in Table 1. The ML algorithms and the grid search are implemented by the v1.1 Scikit-learn library.

Statistical describers were maximum, minimum, mean, median, 1st quartile, 3rd quartile, and SD for each available time series (each component X, Y, and Z was treated as an independent time series). These parameters, referred to as raw data features, were used as discriminators for ML algorithms. The performance evaluation of each ML algorithm was measured after an n-fold cross-validation process, scaling of the time series, and 100 training repetitions with a random ordering of NLBP and CLBP.

The computed SampEn and CF were also included as features in the ML algorithms in a second step. The accuracy and AUC of the ML algorithms were computed using only raw data features and only SampEn or CF. The added value of SampEn and CF in identifying CLBP was assessed.

#### 2.4.3. Most Discriminative Features

Two methods were used to identify the best discriminating feature: Sequential Feature Selector forward (SFS Forward) and Sequential Feature Selector backward (SFS Backward) [30]. The SFS Forward accumulates the best-performing features one by one and creates a hierarchy from the best-performing feature to the worst-performing feature. The SFS Backward starts with all features and removes the worst-performing features one by one. This results in a hierarchy from the longest-remaining feature to the least-remaining feature. Each SFS was run 700 times (7 algorithms × 100 training repetitions).

## 3. Results

### 3.1. SampEn & CF

Four of the 18 calculated SampEn values (six time series and 3 IMUs) showed significant differences between CLBP and NLBP groups. HCF showed significant differences between CLBP and NLBP groups, whereas LCF did not. These statistically significant results are shown in Table 2, while the others have been omitted for simplicity.

### 3.2. Cycle Segmentation

**Figure 3:**
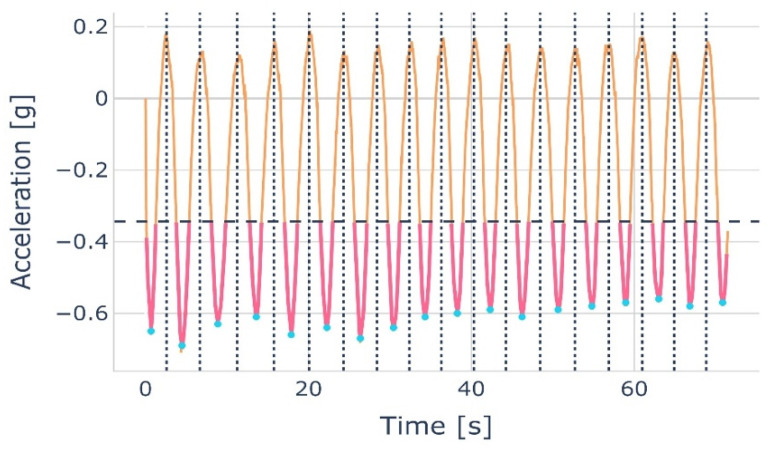
Principle of cycle segmentation based on minima of Acc Z. The dashed horizontal line represents the global threshold (40% above the global minima). The dotted vertical lines represent the cycle limits that lie in the middle of two consecutive local minima. The pink lines show the part of the time series that is below the threshold, the orange lines show the time series that is above the threshold, and the blue dots show the minima of the pink lines.

**Figure 4:**
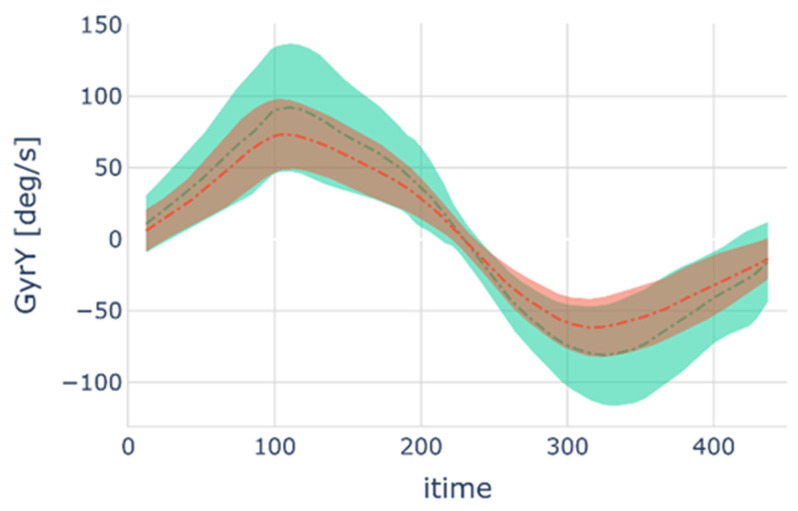
Mean Gyr Y of the SENS2 cycle as function of time is shown for NLBP subjects (green dashed dot line) and for CLBP patients (red dashed dot line). The colored areas correspond to the cycles SD for healthy NLBP subjects (green) and CLBP patients (red or brown when overlapped with green). Note that all cycles were normalized to the same number of points (n = 450) and that mean and SD refer to all cycle values at a given normalized time point (itime).

The result of the segmentation process was a data set of 1678 cycles labeled with their respective index (CLBP patient or healthy NLBP subject). Figure 3 show the principle of segmentation. Figure 4 shows all the computed cycles for Gyr Y of SENS2 as a function of time in the form of a mean cycle and a shaded area indicating the cycle’s SD.

### 3.3. Machine Learning

The optimal hyperparameter values for each ML algorithm are highlighted in bold in Table 1. The performance indicators of all the ML algorithms used are listed in Table 3. 

The best classification accuracy was obtained using the whole sequences with the GaussianNB algorithm. In this case, an accuracy of 79% and an AUC value of 0.85 were obtained. Gaussian naive Bayes process each feature, computing a normal distribution with their mean and standard deviation. To predict the class of a new subject, the algorithm computes the likelihood of the subject’s raw data belonging to the gaussian distribution of NLBP and CLBP. The Gaussian naïve Bayes could compare the scores obtained for each class and decide the best-fitted class for the subject. As the raw data were on a times series, it seems reasonable that the data fit well with the GaussianNB algorithm.

Table 4 shows the effects of using SampEn or CF as features instead of raw IMU data for the whole sequences. The performance globally decreased with SampEn. The best algorithm (GaussianNB) achieved 64% Accuracy and an AUC value of 0.69. Using CF, the best algorithm (SVM RBF) achieved 74% Acc and an AUC value of 0.80. Overall, cycle segmentation produced the worst results, so for simplicity, we have omitted these data from the results table.

### 3.4. Most Discriminative Features

The minimum value of Gyr Y measured by SENS2 was the most discriminative feature (first 355 times in 700 runs), followed by the Q3 of Acc X measured by SENS3. The SD of Acc Y measured by SENS2 was the most discriminating feature of the second test (114 times in 700 runs). The complete results can be found in Table 5.

## 4. Discussion

Our results showed that ML was able to discriminate CLBP patients from NLBP subjects with good accuracy, especially for the GaussianNB algorithm. The identification of the most discriminating features shows that Gyr Y SENS2 min is the best parameter: CLBP patients have a lower flexion angular velocity (in norm) in the return phase of the b&r test than NLBP subjects; the angular velocity was measured by a sensor placed in front of the second sacral vertebra. A wide variety of models for the kinematic analysis of the lumbar region are available in the literature [31]. Several authors have proposed positioning two sensors, one near T12 and the other near S2, to characterize the movements of the lumbar spine in the sagittal plane. Some used this model with an optoelectronic system [32], while others used IMUs with or without comparison to an optoelectronic system [9,11,32,33]. In the positioning of our IMUs, SENS2 (placed at S2) provided the maximum number of discriminative features. SENS1 (placed at T12) alone did not provide us with discriminative data, but the HCF, calculated from SENS2 (S2) and SENS3, did. The optimal number of sensors remains an open discussion, but SENS2 should be preferred for clinical applications. According to our results, the single SENS2 seems to be sufficient to clinically discriminate between CLBP and NLBP subjects; this result may seem attractive for clinical use by reason of its simplicity; however, due to the limited sample of our study, this finding should be treated with caution. Nonetheless, we advocate the use of multiple sensors to analyze lumbopelvic and hip movements in clinical research in order to expand our understanding of the disturbances of these movements in the clinical setting.

Due to the early onset of fatigue, the duration of the b&r test (70 s) could be a source of bias if a CLBP patient is severely physically deconditioned. However, the test can be interrupted after 20 s, and reliable SampEn values can be obtained despite a three-fold shorter time series [20]. Future clinical studies should be conducted to understand the effects of spinal erector muscle fatigue on the b&r test. The use of the b&r test in acute or sub-acute patients has also not been clinically studied.

Some values of SampEn and CF were significantly different between CLBP patients and NLBP subjects when the b&r test was performed. SampEn showed a significant difference for the Gyr Y of SENS1, located at the lower thoracic level, and Gyr Y SENS2, located at the upper sacral level. These differences suggest a lower complexity of variability in angular velocity measured about the transverse axis, which corresponds to the main b&r movement. In other words, CLBP patients show more stereotyped movements of the lumbo–pelvic complex, which is consistent with the optimal variability paradigm of [21,34]: the healthier an individual, the more variability they can show in performing a movement, which is an indication of their adaptability. HCF also showed a significant decrease in CLBP patients, as did SampEn for Acc X of SENS2. In contrast, SampEn for Gyr Z showed an increase. The former describes the complexity of the pelvic tilting movement about an antero–posterior axis, and the latter the complexity of the vertical pelvic displacement, which is an obvious part of the b&r test. As with lumbar muscle activity in a previous study [35], the observed behaviors suggest a tendency toward stereotyped movements in CLBP patients. The literature suggests that the increase or decrease in complexity may indicate two different pathological phenotypes: instability or hypercontrol [36]. It is quite possible that the observed phenomenon of either upward or downward changes in CF is responsible for the less and even non-discriminative results in distinguishing CLBP patients from NLBP participants. In future studies, it would be appropriate to pre-classify CLBP patients using existing relevant clinical tests [37,38,39] to investigate the ability of the b&r test to highlight these two phenotypes.

Despite these observed significant differences, the mean differences between SampEn and HCF in CLBP patients compared with NLBP subjects are so close to those of SEM and MDC that the clinical interest of SampEn can be questioned. Furthermore, although SampEn is easy to compute with any modern computer, a deviation of this parameter from a norm not yet established may be very difficult or impossible to detect by a clinician in practice. It is important to identify more standard kinematic parameters (position, velocity, acceleration) representative of the CLBP population. Visualization of all cycles performed during the b&r test in a single graphical representation has already shown that NLBP participants, for example, achieved overall higher values for angular velocity about the flexion axis than CLBP patients. This component of angular velocity proved to be the most discriminating feature in our study. Therefore, focusing on flexion movement velocity may be a good recommendation for clinicians when managing CLBP patients.

Regardless of the most discriminating features, the ML algorithms were able to accurately identify CLBP patients when performing a b&r test. The algorithm with the best performance is GaussianNB, which is supplied with the whole time series recorded by the IMUs. It achieves an accuracy of 79% and an AUC value of 0.85. It is worth noting that good accuracy can also be achieved when an SVM RBF algorithm is provided with CF from the whole time series. In this case, an accuracy of 74% and an AUC value of 0.80 are achieved. The better performance of the SVM algorithm might be related to a well-known property of this method, which tends to perform better than other ML algorithms when the number of features is small [40].

Abdollahi et al. were able to distinguish LBP patients from NLBP subjects during a kinematic test using ML analysis—specifically SVM—of time series collected via an IMU attached to the sternum [12]. They were also able to classify them according to the risk of chronicity [12]. Similar results with lower accuracy were obtained with the same test but using classical statistical analysis (linear discriminant analysis) [41]. In our study, we found that the SENS2 had higher discriminating power in both classical statistical analysis and ML analysis. However, we did not investigate whether our method is able to classify patients according to their risk of chronicity. The differences between [12] and our study are mainly due to the different experimental protocols. Although we use a sagittal plane test, our test is performed over a longer period of time (60 s of recording) with a sensor recording frequency (100 Hz) five times higher than [12]. The amount of data collected this way allowed us to analyze the complexity of the movement variation. In our study, we did not measure the center of mass motion, and our NLBP population consisted of only 20 subjects, which severely limits the learning ability of the ML process. However, both studies demonstrate the interest in using ML in spinal kinematic analysis to identify and assess CLBP patients. It remains to be shown that these tools are also useful for the therapeutic follow-up of these patients.

As a counterpoint to using biomechanics to better understand LBP conditions, a meta-model created with the help of a panel of 27 multidisciplinary experts is used to illustrate the number of factors that contribute to LBP, disability, quality of life, and other outcomes as well as the number and strength of their interactions [42]. With a problem as multifactorial and complex as LBP, the authors of this counterpoint emphasize the need to integrate the interactions of biopsychosocial factors into research to improve the management of patients with LBP. This suggests that in future works, it will be essential to integrate these biopsychosocial factors and the state of the internal environment into the raw IMU data and the variability indicators computed from them when performing a b&r test to better understand its discriminating power. This may be achieved via analysis from non-invasive portable electrochemical sensors [43] of fluids such as sweat and its relevant components. An ML algorithm can help achieve this goal, as both quantitative and qualitative data can be used.

## 5. Conclusions

The objective of this study was to evaluate the relevance of various machine ML algorithms and SampEn in the identification of LBP conditions. Using the raw data from three IMUs and the SampEn values obtained during a b&r test, the results showed better abilities to discriminate CLBP patients from NLBP subjects for the GaussianNB ML algorithm than the SampEn discriminant values alone. This study demonstrated that: supervised ML and a complexity assessment of trunk movement variability are useful in the identification of CLBP conditions, and that simple kinematic indicators are sensitive to the latter condition.

Regardless of the pathology studied, our study can shed light on the advantages and disadvantages of “standard” statistical analysis and ML-based approaches in clinical applications. In standard statistical analysis, the experimenter/clinician a priori identifies relevant indices and computes them. If the indices are well chosen, they will show statistically significant differences between groups, typically between healthy subjects and patients. By accepting arbitrariness in the choice of indices, such a methodology can show significant results for small populations, typically groups of 20 subjects, which can be easily recruited from a clinician’s patients and acquaintances. One of the advantages of ML is that the algorithm itself finds the most discriminative indices in a non-arbitrary way, but the population has to be larger than in usual statistical analysis, which can make ML complicated to apply in daily clinical practice. What we have shown is that ML may already give good results in discriminating small but accurately selected groups and that it may also identify the main discriminative features of the performed measurements. The latter features might, in turn, be used in quick clinical tests based on the measurement of a single parameter and threshold value. Hence, ML may provide key parameters to be included in more standard approaches already used in daily clinical practice. 

## Figures and Tables

**Table 1:** Classifier’s hyperparameters.

ML Algorithm	Hyperparameters
BF KNN	number of neighbours (**3**, 5, 8, 10), weighting function (uniform, **distance**), algorithm (**Brute-Force** (BF KNN or BF KNN), kd_tree, auto, ball_tree)
Linear SVM	regularization parameter (**0.001**, 0.01, 0.1, 1, 10, 100, 1000)
SVM RBF	C-parameter (0.001, 0.01, 0.1, **1**, 10, 100), kernel coefficient Gamma (**0.001**, 0.01, 0.1, 1, 10, 100)
DT	maximum depth of the tree (1, 5, **10**, 100), function to measure the quality of the splits (**gini**, entropy), strategy to select the split nodes (**best**, random)
RF	maximum depth of the tree (1, 5, **10**, 100), number of trees in the forest (1, 5, 10, **100**), number of features considered in the search for the best split (**1**, 5, 10, 100)
AdaBoost	maximum number of estimators at which boosting stops (5, 10, **50**, 100, 500), weight applied to each classifier at each boosting iteration (0.000001, 0.001, 0.1, **1**, 5, 10, 100)
GaussianNB	ratio of the largest variance of all features added to the variances for computational stability (**0.0000001**, 0.01, 1, 10, 100)

BF KNN: Brute-Force K-Nearest Neighbors, SVM: Support Vector Machine, RBF: radial basis function, DT: Decision Tree, RF: Random Forest, AdaBoost: Adaptive boosting, GaussianNB: Gaussian naive Bayes; hyperparameters in bold are the selected ones by the grid-search function.

**Table 2:** Significative differences (T-test or Wilcoxon test depending on the normality or not of the distribution of the SampEn values) between CLBP and NLBP groups. All parameters are SampEn values defined in Section 2.3.

SampEn	Gyr Y SENS1		Gyr Z SENS2		HCF			Gyr Y SENS2		Acc X SENS2	
	CLBP	NLBP	CLBP	NLBP	CLBP	NLBP		CLBP	NLBP	CLBP	NLBP
Mean	0.161	0.208	0.625	0.516	0.272	0.326	Median	0.217	0.282	0.266	0.389
SD	0.05	0.072	0.168	0.144	0.053	0.100	Q1	0.187	0.220	0.227	0.312
SEM	0.011	0.016	0.038	0.032	0.012	0.022	Q3	0.261	0.343	0.407	0.523
*p*-value	0.021		0.035		0.044		*p*-value	0.021		0.047	
Difference NLBP−CLBP											
Mean	0.035		−0.108		0.055		Mean	−0.034		0.097	
SD	0.168		0.254		0.111		SD	0.159		0.301	
CI	0.074		0.111		0.046		CI	0.070		0.132	
SEM	0.038		0.057		0.024		SEM	0.036		0.067	
MDC	0.104		0.157		0.070		MDC	0.099		0.187	

CI: 95% confidence interval; SEM: Standard Error of Measure; MDC: Minimal Detectable Change; SampEn value for: the Y-axis of the gyroscope from the sensor 1 (Gyr Y SENS1), the Z-axis of the gyroscope from the sensor 2 (Gyr Z SENS2), Hip Complexity Factor (HCF), the Y-axis of the gyroscope from the sensor 2 (Gyr Y SENS2), the X-axis of the accelerometer from the sensor 2 (Acc X SENS2).

**Table 3:** Comparison of prediction performance between the whole sequences and cycle segmentation procedures, for all considered ML algorithms.

	Whole Sequences		Cycle Segmentation	
Algorithms	Accuracy (%)	AUC	Accuracy (%)	AUC
BF KNN	0.63 ± 0.08	0.69 ± 0.09	0.65 ± 0.05	0.67 ± 0.06
Linear SVM	0.72 ± 0.07	0.79 ± 0.07	0.68 ± 0.06	0.71 ± 0.08
SVM RBF	0.52 ± 0.06	0.52 ± 0.09	0.64 ± 0.04	0.71 ± 0.06
DT	0.66 ± 0.08	0.65 ± 0.09	0.66 ± 0.06	0.65 ± 0.06
RF	0.78 ± 0.07	0.83 ± 0.08	0.72 ± 0.05	0.80 ± 0.06
AdaBoost	0.68 ± 0.07	0.74 ± 0.08	0.70 ± 0.06	0.74 ± 0.08
GaussianNB	**0.79 ± 0.08**	**0.85 ± 0.07**	0.69 ± 0.07	0.74 ± 0.07

BF KNN: Brute-Force K-Nearest Neighbors, SVM: Support Vector Machine, RBF: radial basis function, DT: Decision Tree, RF: Random Forest, AdaBoost: Adaptive boosting, GaussianNB: Gaussian Naive Bayes. Bold numbers indicate best prediction results.

**Table 4:** Accuracy and AUC scores for CLBP-NLBP classification using the whole sequences with different features.

Whole Sequences	Raw Data		SampEn		CF	
Algorithms	Accuracy (%)	AUC	Accuracy (%)	AUC	Accuracy (%)	AUC
BF KNN	0.63 ± 0.08	0.69 ± 0.09	0.59 ± 0.10	0.62 ± 0.09	0.73 ± 0.06	0.78 ± 0.06
Linear SVM	0.72 ± 0.07	0.79 ± 0.07	0.53 ± 0.02	0.64 ± 0.10	0.68 ± 0.06	0.74 ± 0.07
SVM RBF	0.52 ± 0.06	0.52 ± 0.09	0.53 ± 0.02	0.64 ± 0.10	**0.74 ± 0.06**	**0.80 ± 0.08**
DT	0.66 ± 0.08	0.65 ± 0.09	0.58 ± 0.07	0.56 ± 0.07	0.60 ± 0.10	0.61 ± 0.10
RF	0.78 ± 0.07	0.83 ± 0.08	0.59 ± 0.08	0.64 ± 0.09	0.68 ± 0.07	0.71 ± 0.07
AdaBoost	0.68 ± 0.07	0.74 ± 0.08	0.55 ± 0.10	0.57 ± 0.10	0.62 ± 0.10	0.62 ± 0.11
GaussianNB	**0.79 ± 0.08**	**0.85 ± 0.07**	**0.64 ± 0.06**	**0.69 ± 0.07**	0.60 ± 0.08	0.60 ± 0.10

BF KNN: Brute-Force K-Nearest Neighbors, SVM: Support Vector Machine, RBF: radial basis function, DT: Decision Tree, RF: Random Forest, AdaBoost: Adaptive boosting, GaussianNB: Gaussian Naive Bayes. Bold numbers indicate best prediction results.

**Table 5:** Number of times the most discriminating characteristics are first and second out of 700 runs.

Feature	First	Feature	Second
Gyr Y SENS2 min	355	Acc Y SENS2 SD	114
Acc X SENS3 Q3	136	Gyr Y SENS2 min	112
Acc Y SENS2 SD	111	Acc X SENS3 Q3	103
Acc Y SENS2 SD	89	Acc Y SENS2 Q1	83
Acc X SENS3 Q1	64	Acc X SENS3 mean	71

Gyr: Gyroscope; Acc: Accelerometer; SENS: Sensor; min: minimum; SD: standard deviation; Q1: 1st quartile; Q3 3rd quartile.

## Data Availability

Data are available at https://osf.io/t4dgr/ (accessed on 19 January 2022), folder “Low Back Pain vs Healthy”. Source codes are available at https://github.com/martinhouryfors/IMU-and-IA-to-Assess-Chronic-Low-Back-Pain (accessed on 15 June 2022).
